# Educational Influences on Late-Life Health: Genetic Propensity and Attained Education

**DOI:** 10.1093/geronb/gbad153

**Published:** 2023-10-20

**Authors:** Malin Ericsson, Brian Finch, Ida K Karlsson, Margaret Gatz, Chandra A Reynolds, Nancy L Pedersen, Miriam A Mosing

**Affiliations:** Department of Medical Epidemiology and Biostatistics, Karolinska Institutet, Stockholm, Sweden; Aging Research Center, Karolinska Institutet & Stockholm University, Stockholm, Sweden; Center for Social and Economic Research, University of Southern California, Los Angeles, California, USA; Department of Medical Epidemiology and Biostatistics, Karolinska Institutet, Stockholm, Sweden; Center for Social and Economic Research, University of Southern California, Los Angeles, California, USA; Department of Psychology, University of California, Riverside, Riverside, California, USA; Department of Medical Epidemiology and Biostatistics, Karolinska Institutet, Stockholm, Sweden; Department of Medical Epidemiology and Biostatistics, Karolinska Institutet, Stockholm, Sweden; Department of Cognitive Neuropsychology, Max Planck Institute for Empirical Aesthetics, Frankurt, Germany; (Social Sciences Section)

**Keywords:** Aging, Educational attainment, Genetics, Gene–environment correlation, Twin studies

## Abstract

**Objectives:**

The educational gradient in late-life health is well established. Despite this, there are still ambiguities concerning the role of underlying confounding by genetic influences and gene-environment (GE) interplay. Here, we investigate the role of educational factors (attained and genetic propensities) on health and mortality in late life using genetic propensity for educational attainment (as measured by a genome-wide polygenic score, PGS_Edu_) and attained education.

**Methods:**

By utilizing genetically informative twin data from the Swedish Twin Registry (*n* = 14,570), we investigated influences of the educational measures, familial confounding as well as the possible presence of passive GE correlation on both objective and subjective indicators of late-life health, that is, the Frailty Index, Multimorbidity, Self-rated health, cardiovascular disease, and all-cause mortality.

**Results:**

Using between-within models to adjust for shared familial factors, we found that the relationship between educational level and health and mortality later in life persisted despite controlling for familial confounding. PGS_Edu_ and attained education both uniquely predicted late-life health and mortality, even when mutually adjusted. Between-within models of PGS_Edu_ on the health outcomes in dizygotic twins showed weak evidence for passive GE correlation (prGE) in the education-health relationship.

**Discussion:**

Both genetic propensity to education and attained education are (partly) independently associated with health in late life. These results lend further support for a causal education-health relationship but also raise the importance of genetic contributions and GE interplay.

Late life is a heterogeneous period with great variability in health, function, and disability. Both, genetic and socioeconomic factors are well-established determinants of this diversity (Ericsson et al., 2019; Madsen et al., 2014; Ostergren, 2018). Observed differences in health across the life span are associated with the level of attained education, where a higher educational level is predictive of better health and a lower mortality risk (Ericsson et al., 2019; Seblova et al., 2020). The importance of education for health can be understood based on its consistent influence on many aspects of life, related to social capital, occupation, income, resources, and health behaviors, among others (Mirowsky & Ross, 2005). Such educational health inequalities remain evident in late life, despite the temporal distance to education and working life. However, whether this relationship is truly causal, that is, higher education may serve as an intervention for reducing health inequalities, or whether underlying confounding factors play a pivotal role, is still poorly understood.

That the level of attained education is substantially influenced by genetic propensities has been clearly demonstrated, both in twin studies (Baier & Lang, 2019; Silventoinen et al., 2017) and more recently in genome-wide association studies (Lee et al., 2018; Okbay et al., 2022; von Stumm et al., 2020). Genetic propensities underlying educational attainment may influence health in late life, presumably through an interplay with the socioeconomic environment over the life course. The interplay between genetic influences, socioeconomic factors, and health starts early in life, with parents passing their predispositions to education and health on to their offspring directly via their genes as well as indirectly by generating a family environment consistent with their own genotypes, which may facilitate health behavior and education. The resulting correlation between offspring genotype and family environment is referred to as passive gene-environment correlation (prGE; Plomin et al., 1977). Susceptibility to environmental influences may also differ depending on genetic predispositions; phenotypic expression is thus influenced by how environmental influences interact with either genetic sensitivity or resilience (Kendler & Baker, 2007). As such, both genetic and environmental effects (including family environment) contribute to socioeconomic and health trajectories directly and potentially via mutual interplay (i.e., gene–environment interaction [GxE] and correlations [rGE]).

Although past genetic research on the relationship between education and health has mostly focused on latent genetic factors using family designs, recent advances in molecular genetics allow for the estimation of individual genetic predispositions. One of the largest genome-wide association meta-analyses to date explored educational attainment in approximately 3 million participants and could reliably identify 3,952 independent associated genetic variants across the genome (Okbay et al., 2022). Based on the resulting summary statistics we can calculate individuals’ genetic propensity for educational attainment in any target sample that is, genome-wide polygenic scores (PGS), by summing the total number of trait-associated alleles across the genome, weighted by their respective association effect size (Choi et al., 2020). Recent work has confirmed the predictive value of a PGS for education (PGS_Edu_), suggesting that childhood SES and PGS_Edu_ are the strongest predictors of educational achievement, especially among the most vulnerable and the most advantaged explaining 14% and 23% of the variance, respectively (von Stumm et al., 2020). Other studies combining genetic and family-based data have shown the importance of genetic influences on the childhood environment in predicting educational success, beyond the individual’s own genetic predisposition (Kong et al., 2018; Willoughby et al., 2021). However, little is known regarding how this relates to the educational gradient in late-life health.

In this study, we aim to investigate the association between education and various late-life health outcomes. Utilizing a unique data set consisting of a large cohort of twins with genetically informative data linked to Swedish health registers, we aim to explore self-reported as well as registry-based health indicators, strengthen causal inferences utilizing family-based methods, and explore familial confounding and gene-environment interplay with conventional PGS_Edu_ analyses extended to a within-family setting (Selzam et al., 2019). For a further in-depth exploration of underlying familial confounding and GE interplay, we additionally investigate genetic propensities (as measured by PGS) to understand individual differences in late-life health (Selzam et al., 2019). Thus, this study increases the understanding of the nature of educational late-life health inequalities and their genetic and environmental components.

Specifically, we aim to:

1) Replicate and extend past findings by estimating causal and familial confounding effects underlying the association between education and a range of late-life health outcomes utilizing a large sample of more than 14,000 twins.
2) Explore the role of gene-environment interplay in the association between education and late-life health inequalities using PGS_Edu_ as a measure of genetic propensity for educational attainment.
3) Investigate the presence of gene-environment correlation (rGE), which could inflate the impact of genetics on the phenotypes or partly explain the education and late-life health associations.

## Materials and Methods

### Data

The Screening Across the Life span of Twins (SALT) study targeted all twins in the Swedish Twin Register (STR) born between 1886 and 1958 and was conducted between 1998 and 2002 via a computer-assisted telephone interview (Lichtenstein et al., 2002, 2006). The data collection consisted of a comprehensive health screening, administered by professional interviewers with medical knowledge. In total 44,919 twins were included in SALT, out of which our final sample consisted of participants with genotype data available to create polygenic scores for educational attainment (*n* = 14,570). Genotyping of the SALT participants is described in the Supplementary Material. All research described was approved by the regional ethics review board and all participants gave informed consent.

### Late-life Health Indicators

To operationalize a comprehensive image of late-life health, several indicators of health were selected. These indicators reflect both objective and subjective health, primarily related to physical aspects of age-related health status.

*The Frailty index (FI)* is constructed from various deficits, related to health, function, and well-being. The FI represents an accumulation of deficits, and the number of health-related items may vary when the index is compiled (Searle et al., 2008). In SALT, a total of 44 items were included to create the FI (Supplementary Table 2). The final index is the individual rate of deficits, that is, calculated as the individual number of deficits divided by the total number of deficits. Details on the FI in SALT can be found elsewhere (Daníelsdóttir et al., 2019; Li et al., 2019). A higher score corresponds to worse health.

*The Cumulative Illness Rating Scale (CIRS)* is a widely applied and validated tool to quantify the burden of diseases in the older population and for assessing physical impairment and multimorbidity (Gatz et al., 2015; Miller et al., 1992). The scale is composed of the number of affected systems, based on both acute and chronic conditions. A higher score indicates a higher degree of multimorbidity.

*Self-rated health (SRH)* has been shown to be a reliable indicator of health and a better predictor of mortality than a doctor’s assessment (Idler & Benyamini, 1997). SRH was measured using a single question; “How do you rate your general health status?” with response alternatives: “excellent,” “very good,” “good,” “fair,” and “poor.” A higher score corresponds to better self-rated health.

*Cardiovascular disease (CVD)* is a generic diagnostic term for diseases that affect the heart and vascular system. It is a leading cause of death in Sweden and internationally. CVD incidence was estimated as the first date of any CVD event (ICD 9: 390-459 and ICD 10: 100-199), occurring from 1960 to the end of 2016. Data were retrieved through linkage with the national patient register.

*All-cause mortality* information was retrieved through linkage with the Swedish population registry. Mortality data (date of death) for the twins in the STR were available up to the end of 2019.

### Socioeconomic Variables

*Educational attainment* was collected through self-report in the SALT data and was harmonized according to the International Standard Classification of Education (ISCED; Eurostat, 2015). The final educational scale had five levels: Primary education (or lower) lower secondary, upper secondary or postsecondary nontertiary, short-cycle tertiary, and university degree (bachelor’s or above).

*Childhood social class* was based on parental occupation retrieved from birth records and self-reports. Childhood social class was coded as a five-level scale in accordance with the Swedish Socioeconomic Index classification: Unskilled manual employees, skilled manual workers, lower nonmanual employees, farmers, self-employed (not including professionals), intermediate nonmanual workers, and higher nonmanual workers (including professionals). Childhood social class was available for a subset of the sample (*n* = 21,948).

Additional covariates were age at interview, birth cohort (in 10-year intervals), sex, zygosity, and smoking. In the analyses where the PGS was included, the 10 first principal components (PCs) were adjusted for. The PCs are a standard method to control for (potentially confounding) genetic ancestry structures in these analyses (Price et al., 2006).

### Statistical Analyses

Analyses were performed in three steps (described in detail subsequently), first investigating familial confounding in the relationship between attained education and the health phenotypes using between-within pair analysis (Begg & Parides, 2003), second, using the polygenic scores to investigate genetic influence on health, and finally applying within-family estimation to the genetic analyses to investigate prGE (Selzam et al., 2019). Separate models were fitted for each of the five different health outcomes. Linear regression was applied to estimate βs for the continuous outcome variables (FI, CIRS, SRH, and educational attainment). Between-within analyses using the PGS_Edu_ were performed using mixed effects models. CVD events and all-cause mortality were analyzed in survival models using Cox proportional hazards models to estimate hazard ratios (HRs). Person-years of follow-up were calculated from the year of the data collection in SALT (1998), until censoring due to emigration, end of the study on December 31, 2019 or, in the models analyzing all-cause mortality, date of death, and in the models investigating CVD’s, date of any CVD event. Age was used as the time scale in all Cox regression models; the HRs were thus adjusted for age. To take into account the dependence within twin pairs in the analyses not applying a within-family approach, a twin-pair identifier was included as a cluster term adjusting the precision of estimates (i.e., standard errors and confidence intervals [Cis]) using robust variances.

### Between-within Analysis of Attained Education and Late-life Health

Between-within twin pair analysis was applied to test for familial confounding in the association between attained education and the health phenotypes. The between-within model is a method used to perform a co-twin control, comparable to a fixed-effects model. In the between-within, model a twin pair mean (between) and a deviation from this mean (within) are analyzed simultaneously in the model. The within-pair estimate is thus adjusted for familial effects (Sjölander et al., 2012). By utilizing information from discordant twin pairs (i.e., twins who differ in exposure and outcome from their co-twin), it is possible to compare population effects to within-pair effects (the effect adjusted for familial confounding). This is because twins are genetically similar (Monozygotic [MZ] twins are identical and Dizygotic [DZ] twins share on average 50% of their co-segregating alleles) and share their rearing environment and intrauterine environment. An attenuation of the within-pair estimate compared with the population estimates indicates the presence of familial confounding. However, if the within-pair estimates are not attenuated in the co-twin control analysis, this would indicate social causation that is, that there is an independent effect of socioeconomic factors on health not explained by other underlying influences (assuming that there are no other confounding factors). In the between-within models of attained education and late-life health outcomes, Model 1 was adjusted for age at interview, sex, and cohort. In Model 2, we additionally included childhood social class to test whether rearing socioeconomic circumstances could explain part of the possible familial confounding. As childhood social class is shared by the twins in a pair, it can only influence the between-pair estimate; however, if the between-pair effect is attenuated, familial factors may be partly attributed to socioeconomic circumstances.

### PGS_Edu_ and Late-life Health

To investigate educational (phenotypic and genetic) influences on late-life health, PGS_Edu_, and attained education were analyzed as the main predictors of the phenotypes in the different regression models. FI, SRH, CIRS, CVD, and all-cause mortality were investigated as outcome variables in all models. Additionally, attained education was analyzed as a function of PGS_Edu_. All models were adjusted for age, the 10 first genetic PCs, sex, and birth cohort. In Model 1, attained education (Model 1.1) and PGS_Edu_ (Model 1.2) were analyzed separately. In Model 2, both education indicators were included simultaneously in the model.

For descriptive purposes, in order to visualize the data and the predictive power of the PGS_Edu_ on attained education and health indicators, we plotted quantile plots. The PGS_Edu_ was divided into 10 quantiles of equal size. Each quantile was then used to predict the phenotype in either a linear (attained education, FI, SRH, and CIRS) or Cox regression (CVD and all-cause mortality) using the median quantile (5) as the reference.

### Between-within Analyses of PGS_Edu_ and Late-life Health

To investigate the role of passive GE correlation (prGE), we estimated the between-within effects of PGS_Edu_ on the various phenotypes in a sample restricted to DZ twins, both same (SSDZ) and opposite sex (OSDZ) twin pairs. Mixed effects models were performed to estimate between and within effects of PGS_Edu_ on our various outcomes. Note that the PGS within DZ twins are only expected to correlate around 0.5 (as DZ twins share 50% of their segregating genes on average), while they share their rearing environment. Hence, the within-pair estimate in this model is adjusted for the shared environment and thus parental genetic influences on the family environment including bias related to assortative mating and population stratification (Selzam et al., 2019). Between-effects are comparable to the estimates performed on the total population that is, non-related individuals. Attenuated within-pair estimates in these models indicate the presence of prGE. In the between-within model, these two effects are included simultaneously in the models and thus are mutually adjusted for. These provide separate fixed effects for the between and the within twin effect. All analyses were adjusted for age at interview, sex, and the first 10 ancestry PC’s. In Model 2, childhood social class was included in order to investigate if the between and within association differed depending on childhood social class, that is, if familial influences could be attributed to childhood social class. As childhood social class is shared within the twin pairs, the within-estimate will not change. However, if the between effect is attenuated and thereby the between-within difference is smaller after adjusting for childhood social class then we can assume that childhood social class is partly the source of the prGE (Selzam et al., 2019), in other words, that the association is due to both childhood social class and genetic predisposition, passively received by the individual.

All analyses were additionally divided by birth cohort, born before 1945 or after, to (arbitrarily) reflect one of the larger educational reforms in Sweden that granted all students access to higher education. The cohort divided analyses on educational (phenotypic and genetic) influences on late-life health were additionally adjusted for smoking (ever smoker/never smoker).

In an additional analysis, we tested the influence of PGS_Edu_ on childhood social class as a further test of rGE, for further details on this statistical model see (Selzam et al., 2019). The presence of rGE can be assumed if there is a statistically significant association. Within-pair correlations for PGS_Edu_ were estimated to investigate the influence of assortative mating where a correlation significantly above 0.5 indicates the presence of assortative mating.

All analyses were conducted using STATA IC version 15 (StataCorp, 2017).

## Results

### Descriptive Analyses

Descriptive data are shown in Supplementary Table 1 for the total sample with genotype data and separately for each sex. Approximately 52% of the study population were women and the sample was skewed towards the later-born cohorts.

### Between-within Analysis of Attained Education and Health

Results from the between-within models of attained education and the various health outcomes using the full twin sample are shown in Table 1. At the between level, equivalent to population-based estimates, a higher attained education predicted better late-life health across health domains (Model 1). By additionally adjusting for childhood social class, within-family estimates will be unaffected while the between-pair estimates may change if childhood social class explains some of the association between education and health. However, the between- and within-pair effect difference did not change notably when childhood social class was added to the models (Model 2). Within-pair estimates were attenuated in all phenotypes, indicating the presence of familial confounding. For FI, SRH, CVD, and all-cause mortality, point estimates were still statistically significant, although attenuated, indicating educational influences were not fully explained by familial factors.

**Table 1.** Results of a Between-within Regression Model of Attained Education Predicting Late-life Health, All Twin Pairs

Education	FI^a^	CIRS	SRH	CVD	All-cause mortality
	β (95% CI)	β (95% CI)	β (95% CI)	HR (95% CI)	HR (95% CI)
Model 1					
Between	−0.70 (−0.79, −0.61)	−0.04 (−0.05, −0.03)	0.12 (0.11, 0.13)	0.93 (0.91, 0.94)	0.89 (0.88, 0.91)
Within	−0.22 (−0.34, −0.10)	0.01 (<−0.01, 0.03)	0.06 (0.05, 0.08)	0.95 (0.93, 0.97)	0.93 (0.90, 1.96)
Within %^b^	31.4	25	50	71.4	63.6
Model 2					
Between	−0.62 (−0.74, −0.50)	−0.04 (−0.05, −0.02)	0.11 (0.09, 0.12)	0.94 (0.91, 0.96)	0.89 (0.86, 0.92)
Within	−0.20 (−0.36, −0.04)	0.02 (<−0.01, 0.04)	0.06 (0.04, 0.08)	0.95 (0.92, 0.98)	0.88 (0.84, 1.94)
Within %^b^	32.3	50	54.6	83.3	No attenuation

*Notes*: CIRS = Cumulative Illness Rating Scale; CVD = cardiovascular disease; FI = Frailty index; SRH = self-rated health.

All analyses are adjusted for age, sex, and cohort. Model 2 is additionally adjusted for childhood social class. Estimates in bold are within the 95% CI. Education: Unit increase and PGS_Edu_: *SD* increase.

Effect sizes are shown as regression betas, except for CVD and mortality where HRs are shown.

^a^Analyzed as percent increase in FI.

^b^Within effect as a percentage of the between effect. CVD and All-cause mortality percentage are based on 1-HR.

### PGS_Edu_ and Late-life Health

**Figure 1.**
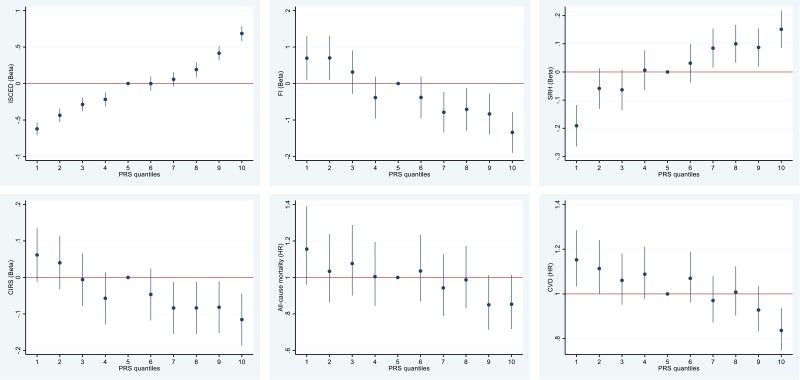
**(A**–**F).** Quantile plot with 10 quantiles of increasing PGS_Edu_ versus attained education (Figure 1**A**) and health phenotypes (Figure 1**B**–**F**; y-axis) using the 5th quantile as the reference in the total sample. A higher PGS_Edu_ predicted a higher educational attainment, less FI, better SRH, less multimorbidity and CVD, and lower mortality.

To test the predictive power of PGS_Edu_ on educational attainment and the health phenotypes we performed quantile regressions. Quantile plots using 10 quintiles and the 5th quantile as a reference value of increasing PGS_Edu_ versus the education and health phenotypes are shown in Figure 1. A higher PGS_Edu_ predicted a higher attained education and better health: lower FI, better SRH, less multimorbidity and CVD, and lower mortality.

Results from analyses investigating the influence of educational factors (genetic and attained) on the health outcomes (not utilizing the family structure of the data) are shown in Table 2. In Model 1, both education and PGS_Edu_ independently predicted the various health outcomes when included separately with higher education and higher PGS_Edu_ being associated with “better” health. In Model 2, where both educational indicators were included simultaneously, the predictive power of both predictors remained, although slightly attenuated as predicted due to the overlap between the two measures.

**Table 2.** Linear and Cox Regression Investigating Late-life Health as a Function of Attained Education and PGS_Edu_, Sample with Genetic Information (PGS_Edu_)

Model	Variable	Education	FI^a^	CIRS	SRH	CVD	All-cause mortality
			β (95% CI)	β (95% CI)	β (95% CI)	HR (95% CI)	HR (95% CI)
Model 1							
*1.1*	Education		−0.50 **(**−0.60, −0.41)	−0.01 (−0.02, <−0.01)	0.10 (0.08, 0.11)	0.95 (0.93, 0.96)	0.93 (0.91, 0.96)
*1.2*	PGS_Edu_	0.34 (0.32, 0.37)	−0.60 (−0.72, −0.47)	−0.05 (−0.07, −0.04)	0.07 (0.06, 0.09)	0.94 (0.91, 0.96)	0.94 (0.90, 0.97)
Model 2							
	Education		−0.42 (−0.52, −0.32)	<−0.01 (−0.02, 0.01)	0.09 (0.08, 0.10)	0.95 (0.94, 0.97)	0.94 (0.91, 0.97)
	PGS_Edu_		−0.45 (−0.58, −0.31)	−0.04 (−0.07, −0.03)	0.04 (0.03, 0.06)	0.95 (0.93, 0.98)	0.96 (0.92. <1.00)

*Notes*: CIRS = Cumulative illness rating scale; CVD = cardiovascular disease; FI = Frailty index; SRH = self-rated health.

Model 1 contains either education (1.1) or PGS (1.2) as a predictor and is adjusted for age, sex, cohort, and the first 10 pc’s with the PGS_Edu_. Model 2 contains both PGS_Edu_ and Education as independent variables. Estimates in bold are within the 95% CI. Education: Unit increase and PGS_Edu_: *SD* increase.

Effect sizes are shown as regression betas, except for CVD and mortality where HRs are shown.

^a^Analyzed as percent increase in FI.

### Between-within Analyses of PGS_Edu_ and Late-life Health

To investigate the presence of prGE, we performed between-within-pair analyses on the relationship between PGS_Edu_ and the different phenotypes utilizing DZ twin pairs (Table 3). Between-pair estimates were comparable to the estimates from the full model including all twins (Table 2, Model 1.2). For all phenotypes, the within-pair association was weaker than the between-pair effect (indicated by lower beta estimates and higher HRs), although the difference was statistically significant only for attained education. The attenuated within-pair estimates for these phenotypes supported the presence of prGE, assortative mating, or population stratification not captured by the PC’s. To test if childhood social class could be the source of prGE we adjusted for this in Model 2. The between-within difference was slightly smaller for education, FI, and SRH compared with Model 1 due to the small attenuation of the between estimates.

**Table 3.** Between-within analyses PGS_Edu_ predicting late-life health in DZ twins (SS and OS)

PGS_Edu_	Education	FI^a^	CIRS	SRH	CVD	All-cause mortality
	β (95% CI)	β (95% CI)	β (95% CI)	β (95% CI)	HR (95% CI)	HR (95% CI)
Model 1						
Between	0.39 (0.36, 0.41)	−0.67 (−0.83, −0.51)	−0.06 (−0.08, −0.04)	0.09 (0.07, 0.11)	0.94 (0.91, 0.97)	0.93 (0.88, 0.97)
Within	0.19 (0.15, 0.24)	−0.13 (−0.47, 0.22)	<0.01 (−0.04, 0.05)	0.05 (<0.01, 0.09)	0.95 (0.90, 1.02)	0.95 (0.85, 1.06)
Within %^b^	48.7	19.4	16.7	55.6	83.3	71.4
Model 2						
Between	0.33 (0.29, 0.36)	−0.48 (−0.68, −0.28)	−0.06 (−0.08, −0.03)	0.07 (0.04, 0.09)	0.93 (0.89, 0.97)	0.88 (0.80, 0.95)
Within	0.19 (0.13, 0.26)	−0.16 (−0.61, 0.30)	−0.01 (−0.07, 0.05)	0.06 (<−0.01, 0.11)	0.98 (0.90, 1.07)	0.96 (0.79, 1.15)
Within %^b^	57.6	33.3	16.7	85.7	28.6	33.3

*Notes*: CIRS = cumulative illness rating scale; CVD = cardiovascular disease; FI = Frailty index; SRH = self−rated health.

All analyses are adjusted for age, sex, cohort, and the first 10 pc’s. Model 2 is additionally adjusted for childhood social class. Estimates in bold are within the 95% CI. Education: Unit increase and PGS_Edu_: *SD* increase.

Effect sizes are shown as regression betas, except for CVD and mortality where HRs are shown.

^a^Analyzed as percent increase in FI.

^b^Within effect as a percentage of the between effect. CVD and All-cause mortality percentage are based on 1-HR.

Additional analyses testing the influence of PGS_Edu_ on childhood social class as a further test of rGE (Supplementary Table 3) showed a statistically significant association, meaning that a genetic predisposition for higher education correlates with childhood rearing environment (i.e., social class), suggesting rGE. To test for assortative mating, we estimated within-pair correlations for PGS_Edu_ in the DZ twins (Supplementary Table 4). Correlation estimates were significantly higher than 0.5 indicating the potential presence of assortative mating.

The additional analyses dividing the sample by cohort (born before 1945 or after) and adjusting for smoking, provided similar findings as in the main analyses (Supplementary Tables 5–7). Estimates indicated slightly stronger educational influences on health in the later-born cohort.

## Discussion

In this study, we explored educational influences on late-life health and mortality, both in terms of genetic propensities and attained education. By combining an epidemiological approach with genetic and twin data and exploring a range of different late-life health indicators, we aimed to replicate and extend past findings on a causal relationship between education and late-life health, and explore genetic influences and the role of prGE.

In line with past research (Mackenbach et al., 2015; Seblova et al., 2020), we observed a clear educational gradient in health, where a higher level of attained education was associated with better health and lower mortality. Twin studies have previously shown that there are familial influences on this relationship, although it may not explain the entire association (Ericsson et al., 2019; Madsen et al., 2010, 2014). Consistent with those reports, we observed a general attenuation of the associations when taking into account familial confounding (within-pair associations). Within-pair estimates provide the effect adjusted for genetic and shared environmental factors within the twin pair, such as childhood socioeconomic circumstances. Attenuation of within-pair associations was most notable for FI and SRH, suggesting that factors shared within families play an important role in the associations between educational attainment and these two health outcomes, respectively. However, within-pair estimates were still significant for FI, SRH, CVD, and all-cause mortality indicating that education has an impact on these health outcomes even when taking familial confounding into account, in line with a causal relationship.

No meaningful difference could be observed when childhood social class was added to the models, indicating that genetic influences or possibly shared environmental factors other than childhood social class likely explain the finding of familial confounding in the education-health associations.

However, the relationship between childhood social class and attained education is intricate, as they partly share genetic influences (Hill et al., 2016; Trzaskowski et al., 2014). Using measured genetic predisposition for educational attainment (PGS_Edu_) enabled us to further explore the nature of genetic confounding and genetic influences on late-life health taking childhood social class into account. Although much attenuated due to the low predictive value of PGS in general (Abdellaoui & Verweij, 2021), we observed significant associations between attained education and late-life health outcomes in line with the phenotypic findings. These findings lend further support to the role of genetic confounding and indicate an overlap between genetic influences on educational attainment and genetic influences on health, also referred to as genetic pleiotropy. Genetic pleiotropy can result from genetic factors on education acting indirectly via the phenotype attained education on health or vice versa (vertical pleiotropy) or that the same genes directly (or via a 3rd phenotype) influence both education and health (horizontal pleiotropy; van Rheenen et al., 2019). The fact that this relationship remained also after adjusting for attained education, could be an indicator that such genetic influences of education on health are at least partly independent of actual attained education (i.e., horizontal pleiotropy).

The relationship between education and health is commonly theorized to originate from environmental factors related to the level of attained education, such as social position, health behaviors, living conditions, and work environment (Breen et al., 2010; Cutler & Lleras-Muney, 2010). Additional years of education, for example through educational reforms expanding compulsory schooling, have been demonstrated to benefit health and function (Lager & Torssander, 2012; Lager et al., 2017). Although together our findings lend further support for the importance of environments associated with higher education, our results broaden this picture by showing that there are genetic factors related to educational success that are connected to better health, independent of actual attained education. These genetic propensities may be related not only to the ability to achieve a higher education but also to factors traditionally linked to a higher attained education, such as positive health behaviors, less risk-taking, more cognitively stimulating activities, and cognitive abilities. However, genetic propensities to education are associated with several other characteristics not directly related to educational attainment, such as personality and psychopathological traits (Krapohl et al., 2014), which also may indirectly be health-promoting. Others have described findings with PGS_Edu_ that are suggestive of passive rGE processes. For example, PGS_Edu_ has been shown to be important for social mobility, where a higher PGS_Edu_ predicts high SES and upward social mobility (Belsky et al., 2018). Children with higher PGS_Edu_ more often come from families with higher socioeconomic status (Belsky et al., 2016) and a higher childhood SES is also a reliable precondition for a high adult SES. This is further supported by findings that, within families, siblings with favorable genetic propensities for socioeconomic factors (income) have better health compared with their less advantaged kin, a relationship that seems to be further enhanced by attained education (Kweon et al., 2020). Our finding that PGS_Edu_ also predicted childhood SES, lends further support for the importance of prGE, meaning not only that children grow up genetically similar to their parents, but also that parents provide a home environment characterized by their genes.

To further explore the potential role of prGE in the relationship between education and health, we compared between- and within associations for PGS_Edu_ and late-life health in dizygotic twins. Attenuated within-pair estimates indicate confounding by prGE (or assortative mating or population stratification). In our results, within effects were somewhat attenuated in all phenotypes. In other words, there was some evidence that prGE (or assortative mating or population stratification) partly explains the education–health relationship. However, between-within associations were only significantly different (CIs were not overlapping) for attained education, suggesting that attained education reflects an intricate interplay between one’s own genetic propensities and contributions to the rearing environment from parental genetic propensities.

Selzam et al. (2019) observed a considerable attenuation in the between-family associations after adjusting for family SES but mainly for cognitive traits. We did not observe a similar attenuation after adjusting for childhood social class in any of our health domains. This means that childhood social class could not account for the attenuated within-pair associations in our sample and instead indicates the influence of other rearing circumstances and possibly assortative mating. A potential role of assortative mating in educational attainment was further supported by our finding of within-pair correlations for PGS_Edu_ above 0.5 in DZ twin pairs, in line with previous findings (Abdellaoui et al., 2015; Hugh-Jones et al., 2016).

The major strengths of this study include the utility of genetically informative family data and the large sample size, allowing us to include a combination of genetic and environmental variables to explore the education-health relationship using a novel approach. Using family-based data to investigate genetic influences also reduces bias related to population structure (Morris et al., 2020). However, this study also has limitations. To comprehensively reflect health later in life, we included several different health measures, where the majority were broader measures of health such as frailty and self-rated health. These most likely have considerable overlap but may also reflect different aspects of health. However, by not using longitudinal health data, we were not able to account for temporal dimensions of health decline. Aging is a time of change and individual differences in health and function can change noticeably over time; in late life most often manifesting as decline. Longitudinal data are crucial to detect health changes. However, even though we were not able to follow health changes over time, we were able to examine several health outcomes in a large population-based data set with a range of ages. Thus, our data consisted of different birth cohorts. As individuals are born into historical contexts that shape life course pathways this may alter patterns of age-related change. Societal change and opportunities, such as access to education and the expansion of the Swedish welfare state play an important role in shaping the life chances of birth cohorts. For example, for birth cohorts born during the first part of the 1900s, access to higher education was restricted for parts of the population (Erikson & Jonsson, 1996). This may mean that the impact of education on health also differs between cohorts. In our analyses, we adjusted for both cohort and age at interview. In additional analyses, we also tested differences between cohorts, but as these are cross-sectional data, it is difficult to draw any reliable conclusions, as earlier-born cohorts were also older when the data were collected. However, our findings were consistent across cohorts, also after adjusting for smoking, but with an indication of stronger educational influences in later-born cohorts, possibly reflecting better educational opportunities in the second part of the 1900s. However, it is also possible that lower educational differences in health in the older cohorts reflect a mortality selection.

A further strength was the inclusion of both self-reported and registry-based health outcomes. However, it is possible that these different types of data may reflect health in different ways. For example, women report poorer health, and men die earlier, commonly referred to as the men–woman health-survival paradox (Alberts et al., 2014). Women’s and men’s socioeconomic opportunities also differ, especially from a historical perspective. These differences were also reflected in our data, where self-reported health was worse among women while the prevalence of CVD and mortality was higher among men. This could also reflect differences between self-reported and registry-based data. However, our results consistently showed a similar pattern across all health outcomes, with higher education being associated with better health and lower incidence of CVD and mortality.

By having access to socioeconomic data from childhood to adulthood, it was possible to investigate whether familial confounding could be attributed to family SES. At the same time, family SES was only available in a subsample, reducing the sample size in family-SES-adjusted analyses. Additionally, there is the possibility of mortality selection influencing the results. Selection due to poor health or mortality is always an issue in studies of the aging population. This selection entails the possibility that a selected sample with lower SES and poorer health is not as likely to survive and be included in the data. The surviving sample may thus be of seemingly better health but is in fact only selected (Zajacova & Burgard, 2013). A selected sample could have attenuated the association between genetic propensities for educational success and better health (Domingue et al., 2017). Last, it is also important to note that attenuated within estimates in a PGS between-within model can not only be the existence of prGE but could also be due to other confounding factors such as assortative mating or population structure, as our results also indicate. The presence of active and reactive GE is also quite plausible in the education-health relationship, but difficult to test with the present design not allowing for further exploration. Twin methods using the between-within approach may also entail issues related to misclassification. This could lead to a biased within-pair estimate, for example withholding a true causal effect in the analyses (Frisell et al., 2012).

To conclude, we found a strong association between educational attainment and all late-life health outcomes, which largely remained evident (though partially attenuated) when adjusting for familial factors, supporting the role of both familial confounding and a causal effect of education on late-life health. In line with this, both attained education and measured genetic propensities for education were partially uniquely related to health in late life, with higher genetic propensities and higher levels of education predicting better health and lower mortality. The fact that genetic propensities were associated with health outcomes over and above attained education may suggest horizontal genetic pleiotropy between educational propensities and later life health, or genetic effects via other positive health-related behaviors independent of attained education. Additionally, we observed a possible influence of prGE in the education-health relationship. In summary, our results strengthen the causal influence of education on late-life health but also emphasize the importance of shared genetic propensities on both education and health and together suggest an intricate interplay between genes and environment both over the life course and across generations.

## Supplementary Material

gbad153_suppl_Supplementary_Tables_S1-S7
