# Supplementary material for: Educational Influences on Late-Life Health: Genetic Propensity and Attained Education
Source: J Gerontol B Psychol Sci Soc Sci. 2023 Oct 20;79(1):gbad153. doi: 10.1093/geronb/gbad153 (PMC10745256; doi:10.1093/geronb/gbad153)
Supplement: gbad153_suppl_Supplementary_Tables_S1-S7 [file gbad153_suppl_supplementary_tables_s1-s7.docx]

# ***The Journals of Gerontology, Series B: Psychological Sciences and Social Sciences* Online Supplementary Material: Malin Ericsson, Brian Finch, Ida K Karlsson, Margaret Gatz, Chandra A Reynolds, Nancy L Pedersen, & Miriam Mosing. Educational influences on late-life health: Genetic propensity and attained education.**

# Genotype information in SALT

Genotype information for the participants in the SALT study is based on two separate waves: TwinGene and SALTY (Zagai et al., 2019). As part of the TwinGene project conducted between 2004 and 2008, blood-based DNA was extracted in 9,896 participants, including all available dizygotic (DZ) twins and one twin from each available monozygotic (MZ) twin pair, and genotyped in 2009 and 2010 using the 700K Illumina OmniExpress bead chip. As part of the SALTY study, saliva-based DNA was collected in an additional 5,004 younger participants in the SALT study in 2009/2010 and was genotyped using the Illumina 550K PsychArray BeadChip in 2014 and 2015 (Zagai et al., 2019). Stringent QC procedures were applied to the genotype data and PGS were constructed separately for the two waves of genotyping (platforms used). Data were imputed against the Haplotype Reference Consortium (HRC) imputation panels and markers were restricted to the SNPs present in the third phase of the international HapMap project (HapMap 3). The PGS_Edu_ used weights from the most recent GWAS for education (Okbay et al., 2022), after accounting for linkage disequilibrium (correlation of nearby SNPs) with LDpred2 (Privé et al., 2021). For further details on the QC procedure and PRS calculation see Becker et al. (2021).

**eTable 1. Descriptive statistics of the SALT sample with available genotype data.**

|  | **All** | | **Men** | | **Women** | |
| --- | --- | --- | --- | --- | --- | --- |
| n | 14,570 |  | 6,931 |  | 7,639 |  |
| **Zygosity^1^**  n (%) |  |  |  |  |  |  |
| MZ | 2,520 | 17.30 | 1,252 | 18.06 | 1,268 | 16.60 |
| SSDZ | 6,029 | 41.38 | 2,767 | 39.92 | 3,262 | 42.70 |
| OSDZ | 6,019 | 41.31 | 2,912 | 42.01 | 3,107 | 40.67 |
| Unknown | 2 | 0.01 | - |  | 2 | 0.03 |
| **Birth cohort**  n (%) |  |  |  |  |  |  |
| 1911-1930 | 1,318 | 9.05 | 670 | 9.67 | 648 | 8.48 |
| 1931-1940 | 2,933 | 20.13 | 1,491 | 21.51 | 1,442 | 18.88 |
| 1941-1950 | 6,328 | 43.43 | 2,969 | 42.84 | 3,359 | 43.97 |
| 1951-1958 | 3.991 | 27.39 | 1,801 | 25.98 | 2,190 | 28.67 |
| **Age at interview** |  |  |  |  |  |  |
| Mean, SD | 55.44 | 8.09 | 55.84 | 8.11 | 55.22 | 8.10 |
| Ever smoker |  |  |  |  |  |  |
| n (%) |  |  |  |  |  |  |
| Yes | 8,688 | 59.8 | 4,304 | 62.3 | 4,384 | 57.5 |
| **PGS_Edu_^b^** |  |  |  |  |  |  |
| Mean, SD | 0.001 | 1.00 | 0.03 | 1.00 | -0.03 | 1.00 |
| **Education (ISCED)** |  |  |  |  |  |  |
| (Mean, SD) | 1.82 | 1.39 | 1.76 | 1.38 | 1.87 | 1.40 |
| **Childhood social class** |  |  |  |  |  |  |
| (Mean, SD) | 1.06 | 1.14 | 1.06 | 1.14 | 1.07 | 1.14 |
| **Frailty (FI)** |  |  |  |  |  |  |
| (Mean, SD) | 0.12 | 0.08 | 0.11 | 0.07 | 0.13 | 0.09 |
| **Self-rated health (SRH)** |  |  |  |  |  |  |
| (Mean, SD) | 1.95 | 0.95 | 1.86 | 0.90 | 2.03 | 0.99 |
| **Multimorbidity CIRS** |  |  |  |  |  |  |
| (Mean, SD) | 1.01 | 0.99 | 0.92 | 0.97 | 1.09 | 1.01 |
| **Cardiovascular disease/event**  n (%) |  |  |  |  |  |  |
| Yes | 5,836 | 40.05 | 3,121 | 45.03 | 2,715 | 35.54 |
| **Deceased**  n (%) |  |  |  |  |  |  |
| Yes | 2,458 | 16.87 | 1,412 | 20.37 | 1,046 | 13.69 |

Notes. FI = Frailty index, CIRS = Cumulative Illness Rating Scale, SRH = self-rated health, and CVD = cardiovascular disease.

^1^ MZ=Monozygotic, SSDZ=Same-sex dizygotic, and OSDZ=Opposite-sex dizygotic

^b^ Polygenic scores PGS_Edu_ are presented as standardized z-scores

**eTable 2. List of variables to create the Frailty Index (FI) in SALT, modified from Li et al. (2019)**

| Allergy | Glandular diseases (excluding goiter) | Neck pain |
| --- | --- | --- |
| Angina pectoris | Goiter | Number of serious infections/year (not respiratory) |
| Asthma | Gout | Osteoporosis |
| Back pain | Health status prevents you from doing things you want to do | Physical handicap |
| Buzzing in the ears | Hearing | Recurrent periods of coughing |
| Cancer, tumor disease or leukemia | Heart attack | Recurring urinary tract problems |
| Crohn's disease or Ulcerative colitis | Heart failure | Rheumatoid arthritis |
| Chronic lung disease | High blood pressure | Sciatica |
| Diabetes | Hip joint problem | Stomach or intestine problems |
| Dizziness | Irregular cardiac rhythm/atrial fibrillation | Stroke |
| Felt depressed | Kidney disease | TIA attacks |
| Felt happy | Knee joint problem | Vascular spasm in the legs |
| Felt lonely | Lipid disorder | Venous thrombosis |
| Gall bladder problem | Liver disease | Vision |
| General health | Migraine |  |

**eTable 3. Linear regression investigating PGS_Edu_ as a predictor of childhood social class all twins.**

|  | **Childhood social class** | |
| --- | --- | --- |
| PGS_Edu_ | **0.23** | 0.20, 0.26 |

**eTable 4. Intra-pair correlations of PGS_Edu_, DZ twins.**

|  | **DZ**  **(all)** | **SSDZ** | **OSDZ** |
| --- | --- | --- | --- |
| PGS_Edu_ | **.55** | **.55** | **.55** |
| Attained education | **.48** | **.51** | **.45** |

*Note.* Bold estimates indicate correlations significantly different from 0.5.

**eTable 5. Results of a between-within regression model of attained education predicting late-life health, divided by birth cohort (all twin pairs). Effect sizes are shown as regression betas, except for CVD and mortality where HRs are shown.**

| **Education** | **FI^a^** | **CIRS** | **SRH** | **CVD** | **All-cause mortality** |
| --- | --- | --- | --- | --- | --- |
|  | β (95% CIs) | β (95% CIs) | β (95% CIs) | HR (95% CIs) | HR (95% CIs) |
| **Birth cohort 1911-1945** | | |  |  |  |
| **Model 1** |  |  |  |  |  |
| Between | **-0.61** (-0.73, -0.50) | **-0.03** (-0.04, -0.02) | **0.11** (0.10, 0.12) | **0.92** (0.90, 0.93) | **0.91** (0.89, 0.93) |
| Within | -0.11 (-0.26, 0.05) | 0.02 (<-0.01, 0.04) | **0.06** (0.04, 0.08) | **0.95** (0.93, 0.97) | **0.92** (0.89, 1.95) |
|  |  |  |  |  |  |
| **Model 2** |  |  |  |  |  |
| Between | **-0.48** (-0.67, -0.28) | -0.02 (-0.04, <0.01) | **0.10** (0.07, 0.12) | **0.93** (0.90, 0.95) | **0.91** (0.86, 0.92) |
| Within | -0.09 (-0.32, 0.14) | 0.02 (-0.01, 0.05) | **0.06** (0.01, 0.07) | **0.95** (0.92, 0.99) | **0.88** (0.84, 0.94) |
| **Birth cohort 1946-1958** | | |  |  |  |
| **Model 1** |  |  |  |  |  |
| Between | **-0.86** (-0.99, -0.73) | **-0.06** (-0.08, -0.05) | **0.13** (0.12, 0.15) | **0.91** (0.88, 0.94) | **0.85** (0.88, 0.95) |
| Within | **-0.38** (-0.57, -0.19) | <0.01 (<-0.03, 0.02) | **0.07** (0.05, 0.09) | **0.95** (0.91, 0.99) | **0.88** (0.83, 0.94) |
|  |  |  |  |  |  |
| **Model 2** |  |  |  |  |  |
| Between | **-0.77** (-0.93, -0.61) | **-0.06** (-0.08, -0.04) | **0.11** (0.09, 0.13) | **0.93** (0.89, 0.96) | **0.89** (0.80, 0.93) |
| Within | **-0.28** (-0.50, -0.07) | 0.01 (-0.02, 0.04) | **0.07** (0.04, 0.09) | **0.94** (0.89, 0.99) | **0.88** (0.79, 0.98) |

*Note.* All analyses are adjusted for age, sex, and cohort. Model 2 is additionally adjusted for childhood social class. Estimates in bold are within the 95% CI While estimates for FI and SRH are regression betas, all other estimates are ORs or HRs. Education: unit increase and PGS_Edu_: SD increase.

^a^ Analyzed as percent increase in FI

**eTable 6. Linear and Cox regression investigating late-life health as a function of attained education and PGS_Edu_, divided by birth cohort (sample with genetic information (PGS_Edu_)). Effect sizes are shown as regression betas, except for CVD and mortality where HRs are shown.**

|  |  | **Education** | **FI^a^** | **CIRS** | **SRH** | **CVD** | **All-cause mortality** |
| --- | --- | --- | --- | --- | --- | --- | --- |
|  |  |  | β (95% CIs) | β (95% CIs) | β (95% CIs) | HR (95% CIs) | HR (95% CIs) |
| **Birth cohort 1911-1945** | | |  |  |  |  |  |
| *Model 1* |  |  |  |  |  |  |  |
| *1.1* | Education |  | **-0.46 (-**0.59, -0.33) | <-0.01 (-0.02, 0.01) | **0.09** (0.09, 0.10) | **0.94** (0.92, 0.96) | **0.94** (0.91, 0.96) |
|  |  |  |  |  |  |  |  |
| *1.2* | PGS_Edu_ | **0.34** (0.31, 0.37) | **-0.50 -**(0.70, -0.31) | **-0.04** (-0.07, -0.02) | **0.06** (0.04, 0.08) | **0.94** (0.91, 0.96) | **0.95** (0.91, 0.99) |
| *Model 2* |  |  |  |  |  |  |  |
|  | Education |  | **-0.39** (-0.53, -0.26) | <0.01 (-0.01, 0.02) | **0.08** (0.06, 0.09) | **0.95** (0.94, 0.97) | **0.94** (0.91, 0.97) |
|  | PGS_Edu_ |  | **-0.36** (-0.56, -0.17) | **-0.04** (-0.07, -0.02) | **0.03** (0.01, 0.06) | **0.95** (0.93, 0.98) | 0.97 (0.93. 1.02) |
| **Adjusted for smoking** | | |  |  |  |  |  |
| *Model 1* | |  |  |  |  |  |  |
| *1.1* | Education |  | **-0.46 (-**0.59, -0.33) | <-0.01 (-0.02, 0.01) | **0.09** (0.07, 0.10) | **0.94** (0.92, 0.96) | **0.93** (0.90, 0.96) |
|  |  |  |  |  |  |  |  |
| *1.2* | PGS_Edu_ | **0.35** (0.32, 0.38) | **-0.46 -**(0.65, -0.26) | **-0.04** (-0.06, -0.01) | **0.06** (0.03, 0.08) | **0.96** (0.93, 0.99) | 0.96 (0.92, >1.00) |
| *Model 2* |  |  |  |  |  |  |  |
|  | Education |  | **-0.41** (-0.54, -0.27) | <0.01 (-0.01, 0.02) | **0.08** (0.06, 0.09) | **0.94** (0.92, 0.96) | **0.93** (0.90, 0.96) |
|  | PGS_Edu_ |  | **-0.31** (-0.51, -0.11) | **-0.04** (-0.06, -0.01) | **0.03** (0.01, 0.06) | 0.98 (0.95, 1.01) | 0.98 (0.94. 1.03) |
| **Birth cohort 1946-1958** | | |  |  |  |  |  |
| *Model 1* |  |  |  |  |  |  |  |
| *1.1* | Education |  | **-0.57 (-**0.71, -0.43) | **-0.03** (-0.04, -0.01) | **0.11** (0.10, 0.13) | **0.93** (0.90, 0.96) | **0.90** (0.83, 0.97) |
|  |  |  |  |  |  |  |  |
| *1.2* | PGS_Edu_ | **0.34** (0.31, 0.37) | **-0.67 -**(0.85, -0.50) | **-0.06** (-0.08, -0.04) | **0.09** (0.06, 0.11) | **0.94** (0.91, 0.96) | **0.86** (0.78, 0.96) |
| *Model 2* |  |  |  |  |  |  |  |
|  | Education |  | **-0.46** (-0.61, -0.32) | <-0.02 (-0.03, <0.01) | **0.10** (0.08, 0.11) | **0.95** (0.94, 0.97) | 0.93 (0.85, >1.00) |
|  | PGS_Edu_ |  | **-0.51** (-0.61, -0.32) | **-0.05** (-0.08, -0.03) | **0.05** (0.03, 0.07) | **0.92** (0.88, 0.97) | **0.89** (0.79, 0.99) |
| **Adjusted for smoking** | | |  |  |  |  |  |
| *Model 1* |  |  |  |  |  |  |  |
| *1.1* | Education |  | **-0.54 (-**0.68, -0.40) | **-0.03** (-0.04, -0.01) | **0.10** (0.09, 0.12) | **0.93** (0.90, 0.97) | **0.91** (0.84, 0.99) |
|  |  |  |  |  |  |  |  |
| *1.2* | PGS_Edu_ | **0.33** (0.31, 0.36) | **-0.62 -**(0.80, -0.45) | **-0.06** (-0.08, -0.04) | **0.08** (0.06, 0.10) | **0.91** (0.87, 0.95) | **0.88** (0.79, 0.98) |
| *Model 2* |  |  |  |  |  |  |  |
|  | Education |  | **-0.44** (-0.58, -0.30) | -0.02 (-0.03, <0.01) | **0.09** (0.08, 0.11) | **0.95** (0.91, 0.98) | 0.93 (0.86, 1.01) |
|  | PGS_Edu_ |  | **-0.48** (-0.66, -0.29) | **-0.05** (-0.08, -0.03) | **0.05** (0.03, 0.07) | **0.93** (0.88, 0.97) | 0.90 (0.81. 1.01) |

*Note.* Model 1 contains either education (1.1) or PGS (1.2) as a predictor and is adjusted for age, sex, cohort, and the first 10 pc’s with the PGS_Edu_. Model 2 contains both PGS_Edu_ and Education as independent variables. Estimates in bold are within the 95% CI. Education: unit increase and PGS_Edu_: SD increase.

^a^ Analyzed as percent increase in FI

**eTable 7.** **Between-within analyses PGS_Edu_ predicting late-life health in DZ twins (SS and OS), divided by birth cohort. Effect sizes are shown as regression betas, except for CVD and mortality where HRs are shown.**

| **PGS_Edu_** |  | **Education** | **FI^a^** | **CIRS** | **SRH** | **CVD** | **All-cause mortality** |
| --- | --- | --- | --- | --- | --- | --- | --- |
|  |  | β (95% CIs) | β (95% CIs) | β (95% CIs) | β (95% CIs) | HR (95% CIs) | HR (95% CIs) |
| **Birth cohort 1911-1945** | | | |  |  |  |  |
| **Model 1** |  |  |  |  |  |  |  |
| Between |  | **0.41** (0.37, 0.5) | **-0.55** (-0.80, -0.31) | **-0.04** (-0.07, -0.01) | **0.07** (0.04, 0.10) | **0.95** (0.92, 0.98) | **0.94** (0.89, 0.99) |
| Within |  | **0.19** (0.12, 0.25) | -0.33 ( -0.79, 0.14) | -0.04 (-0.11, 0.02) | 0.05 (-0.01, 0.11) | 0.95 (0.88, 1.02) | 0.96 (0.85, 1.09) |
|  |  |  |  |  |  |  |  |
| **Model 2** |  |  |  |  |  |  |  |
| Between |  | **0.36** (0.30, 0.42) | **-0.48** (-0.84, -0.13) | **-0.05** (-0.09, <-0.01) | **0.07** (0.03, 0.12) | 0.94 (0.89, 1.00) | **0.88** (0.79, 0.97) |
| Within |  | **0.20** (0.09, 0.31) | -0.36 (-1.05, 0.34) | -0.08 (-0.18, 0.02) | 0.06 (-0.02, 0.15) | 0.96 (0.85, 1.07) | 1.01 (0.80, 1.26) |
| **Birth cohort 1946-1958** | | | |  |  |  |  |
| **Model 1** |  |  |  |  |  |  |  |
| Between |  | **0.36** (0.33, 0.40) | **-0.77** (-0.99, -0.56) | **-0.08** (-0.10, -0.05) | **0.10** (0.07, 0.13) | **0.91** (0.86, 0.96) | **0.86** (0.76, 0.98) |
| Within |  | **0.20** (0.14, 0.27) | -0.15 ( -0.37, 0.67) | 0.06 (-0.01, 0.13) | 0.04 (-0.02, 0.11) | 0.98 (0.87, 1.09) | 0.88 (0.65, 1.17) |
|  |  |  |  |  |  |  |  |
| **Model 2** |  |  |  |  |  |  |  |
| Between |  | **0.31** (0.27, 0.35) | **-0.49** (-0.74, -0.25) | **-0.07** (-0.09, -0.04) | **0.07** (0.04, 0.10) | **0.93** (0.87, <1.00) | 0.87 (0.75, 1.01) |
| Within |  | **0.19** (0.11, 0.27) | -0.02 (-0.62, 0.59) | 0.04 (-0.03, 0.12) | 0.05 (-0.02, 0.12) | 0.99 (0.86, 1.13) | 0.84 (0.59, 1.18) |

*Note.* All analyses are adjusted for age, sex, cohort, and the first 10 pc’s. Model 2 is additionally adjusted for childhood social class. Estimates in bold are within the 95% CI.

PGS_Edu_: SD increase.

^a^ Analyzed as percent increase in FI.

# **References**

Becker, J., Burik, C. A. P., Goldman, G., Wang, N., Jayashankar, H., Bennett, M., Belsky, D. W., Karlsson Linnér, R., Ahlskog, R., Kleinman, A., Hinds, D. A., Agee, M., Alipanahi, B., Auton, A., Bell, R. K., Bryc, K., Elson, S. L., Fontanillas, P., Furlotte, N. A., . . . andMe Research, G. (2021). Resource profile and user guide of the Polygenic Index Repository. *Nature Human Behaviour*. https://doi.org/10.1038/s41562-021-01119-3

Li, X., Ploner, A., Karlsson, I. K., Liu, X., Magnusson, P. K. E., Pedersen, N. L., Hägg, S., & Jylhävä, J. (2019). The frailty index is a predictor of cause-specific mortality independent of familial effects from midlife onwards: a large cohort study [journal article]. *BMC Medicine*, *17*(1), 94. <https://doi.org/10.1186/s12916-019-1331-8>

Okbay, A., Wu, Y., Wang, N., Jayashankar, H., Bennett, M., Nehzati, S. M., Sidorenko, J., Kweon, H., Goldman, G., Gjorgjieva, T., Jiang, Y., Hicks, B., Tian, C., Hinds, D. A., Ahlskog, R., Magnusson, P. K. E., Oskarsson, S., Hayward, C., Campbell, A., . . . LifeLines Cohort, S. (2022). Polygenic prediction of educational attainment within and between families from genome-wide association analyses in 3 million individuals. *Nature Genetics*, *54*(4), 437-449. <https://doi.org/10.1038/s41588-022-01016-z>

Privé, F., Arbel, J., & Vilhjálmsson, B. J. (2021). LDpred2: better, faster, stronger. *Bioinformatics*, *36*(22-23), 5424-5431. <https://doi.org/10.1093/bioinformatics/btaa1029>

Zagai, U., Lichtenstein, P., Pedersen, N. L., & Magnusson, P. K. E. (2019). The Swedish Twin Registry: Content and Management as a Research Infrastructure. *Twin Res Hum Genet*, *22*(6), 672-680. <https://doi.org/10.1017/thg.2019.99>
